# Supplementary material for: The microbiome biomarkers of pregnant women’s vaginal area predict preterm prelabor rupture in Western China
Source: Front Cell Infect Microbiol. 2024 Oct 31;14:1471027. doi: 10.3389/fcimb.2024.1471027 (PMC11560878; doi:10.3389/fcimb.2024.1471027)
Supplement: Supplementary file 1 [file DataSheet1.zip › compare_1/Community/Pieplot/species/sample/P28.species.pieplot.pdf]

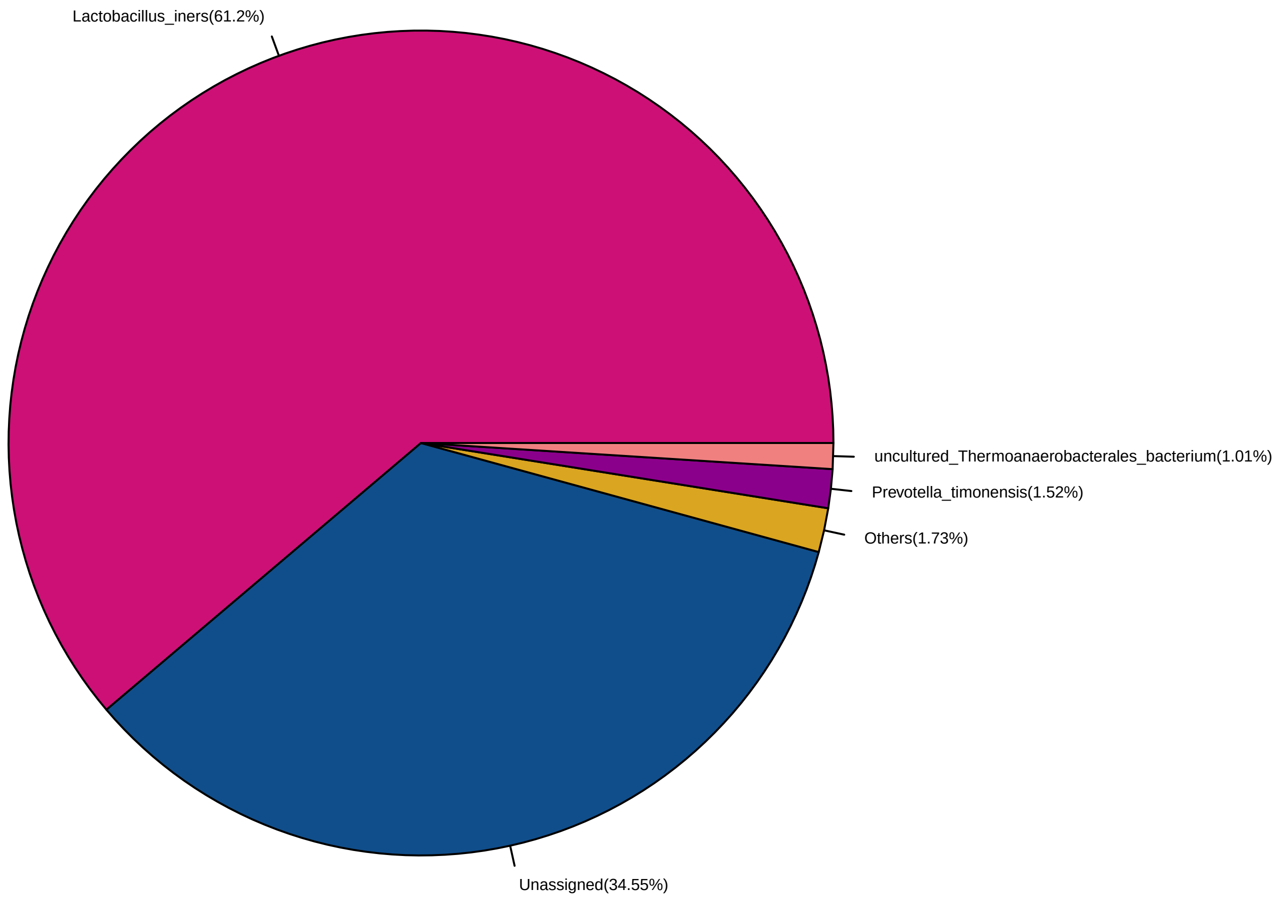

Lactobacillus_iners(61.2%)
uncultured_Thermoanaerobacterales_bacterium(1.01%)
Prevotella_timonensis(1.52%)
Others(1.73%)
Unassigned(34.55%)
